# Factors associated with the progression of mesangial lesions in IgA nephropathy: A comparative analysis of renal re-biopsies

**DOI:** 10.3389/fendo.2022.1004289

**Published:** 2022-11-21

**Authors:** Yetong Li, Shimin Jiang, Hongmei Gao, Yue Yang, Xiaorong Liu, Wenge Li

**Affiliations:** ^1^ Department of Nephrology, Beijing Children’s Hospital, National Center for Children’s Health, Capital Medical University, Beijing, China; ^2^ Department of Nephrology, China-Japan Friendship Hospital, Beijing, China

**Keywords:** IgA nephropathy, renal re-biopsies, mesangial lesions, renal-angiotensin system receptors, glomerular fibrosis

## Abstract

**Objectives:**

IgA nephropathy (IgAN) is the most common primary glomerular disease, and is the leading cause of chronic renal failure. Because mesangial lesions are the main pathological changes seen in IgAN, we investigated factors associated with the progression of mesangial lesions in IgAN.

**Methods:**

We enrolled participants with IgAN who underwent repeat renal biopsies. Based on the progression of mesangial proliferative lesions, the participants were divided into progressive and stable groups. The progression group included participants with a ratio of mesangial cell proliferation score ≥ 1.1 (i.e., proliferation of > 10%) in the second biopsy specimen compared to the first biopsy specimen. The stable group included participants who did not fulfill the aforementioned criteria. We recorded the laboratory parameters, expression of renin-angiotensin system (RAS) receptors (angiotensin II type 1 receptor [AT1R], angiotensin II type 2 receptor [AT2R], Mas receptor [MasR], and the Mas-related G protein-coupled receptor, member D [MrgD]) and mesangial matrix proteins (collagen [Col] IV, fibronectin [FN] and laminin) at the first and second renal biopsies, and the use of immunosuppressive therapy and/or RAS blockers after the first biopsy.

**Results:**

We enrolled 24 patients with IgAN who underwent repeat renal biopsies. Half of patients showed progression of mesangial lesions on repeat renal biopsy after a median of 4.3 (1–6) years. The progression group had significantly higher expression levels of AT1R and mesangial matrix proteins (Col IV and FN), and significantly lower expression of AT2R and MasR, compared to the stable group. Multivariate analysis showed that the use of RAS blockers (hazard ratio [HR], 0.27; 95% CI, 0.08–0.97; p < 0.05) and the level of proteinuria (HR, 1.8; 95% CI, 1.04–3.12; p < 0.05) were associated with progression of mesangial lesions. Additionally, the progression group exhibited a more rapid decline of renal function compared to the stable group (0.38 and 0.012 ml/min/1.73 m^2^/month, respectively; p = 0.004).

**Conclusions:**

Continuous activation of the intrarenal RAS and massive proteinuria correlate with histological progression of mesangial lesions in IgAN patients, which may further accelerate the deterioration of renal function.

## Introduction

IgA nephropathy (IgAN) is a common primary glomerular disease. Patients with IgAN experience a slow but relentless clinical course that may lead to end-stage kidney disease (1). Almost 30% of patients require dialysis or transplant within 20 years after diagnosis. Mesangial cell hyperplasia and mesangial matrix accumulation (i.e., mesangial lesions) are the main pathological lesions of IgAN, and directly cause glomerulosclerosis and, subsequently, renal failure (2, 3).

The renin-angiotensin system (RAS) plays an important role in the pathophysiology of renal diseases. IgAN is characterized by local activation of the RAS and upregulated expression of the angiotensin receptor type 1 (AT1R) (3–5). New components of the RAS have been identified recently, including angiotensin (Ang)-(1-7), Mas receptor (MasR), Ang-(1-9) and alamandine (6, 7). Alamandine is a vasoactive peptide with similar protective actions to Ang-(1-7); it acts through the Mas-related G protein-coupled receptor, member D (MrgD). In experimental models, the newly identified components exhibited vasodilatory, cytostatic, anti-thrombotic, anti-inflammatory, and anti-fibrotic effects. Binding to RAS receptors activates the RAS pathway and other sequential cascades that damage mesangial cells and glomerular structures, eventually leading to terminal fibrosis (8, 9).

Furthermore, clinical examination cannot accurately identify the histopathological findings of interest. We investigated changes in pathological findings on repeat renal biopsies. Although the pathogenesis of IgAN has been explored previously, factors associated with the progression of mesangial lesions have not been explored. We evaluated the progression of mesangial lesions in IgAN patients who underwent two renal biopsies to determine whether there is a correlation of intrarenal RAS receptors with the progression of mesangial lesions, as well as the effect of progression of mesangial lesions on the decline of renal function.

## Patients and methods

### Study population

We enrolled IgAN patients aged ≥ 18 years who underwent two renal biopsies at our hospital between January 2001 and December 2020. We included biopsy-proven IgAN patients who underwent two renal biopsies at an interval of 1–6 years, were aged 18–60 years at the time of repeat renal biopsy, and had an estimated glomerular filtration rate (eGFR) of > 15 ml/min/1.73 m^2^ at the time of the first renal biopsy. We excluded patients with secondary IgAN, other renal diseases (such as purpuric nephritis, cirrhosis, lupus nephritis, diabetic nephropathy, malignant hypertension, and malignant tumors), or missing clinical data.

### Data collection

We reviewed the medical records and extracted the age, sex, body mass index, and laboratory parameters, including serum albumin and creatine levels, hematuria (≥ 5 red blood cells per high-power field), and proteinuria. The eGFR was calculated using the creatinine-based Chronic Kidney Disease Epidemiology Collaboration equation (10). Mean arterial pressure was calculated as the diastolic pressure plus one-third of the pulse pressure.

### Progression of mesangial lesions on renal biopsy

**Figure 1:**
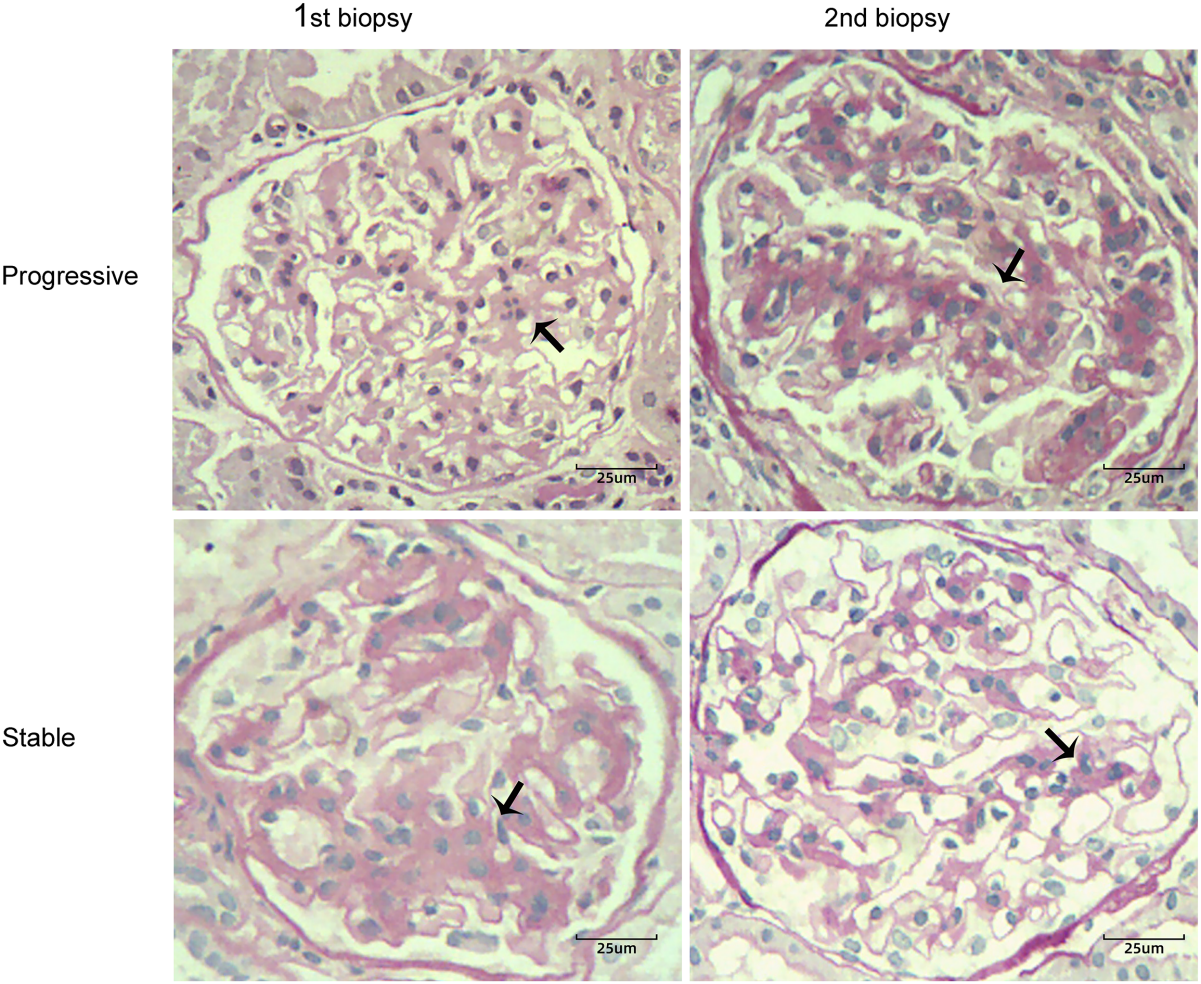
Two cases of IgA nephropathy with different degrees of mesangial proliferation (black arrow) between the first and second renal biopsies (periodic acid-Schiff stain, ×400). Case 1 showed progression of mesangial hyperplasia in the second biopsy specimen; Case 2 showed regression of mesangial lesions in the second biopsy specimen.

Renal biopsy specimens were analyzed using light microscopy, immunofluorescence, and electron microscopy. According to the Oxford score, mesangial proliferation was defined as ≥ 4 mesangial cell nuclei per mesangial area in glomeruli (11–13). Mesangial hypercellularity was categorized as grade 0–3 for < 4, 4–5, 6–7, ≥ 8 mesangial cells/mesangial area, respectively. The mesangial hypercellularity score was based on the percentage of glomeruli showing the most severe mesangial hypercellularity; this was used because it is more reproducible. Progression of mesangial hyperplasia was defined as a ratio of mesangial cell proliferation scores between the first and second renal biopsies of > 1.1 (i.e., hyperplasia > 10%). The participants were divided into progressive (ratio > 1.1; hyperplasia > 10%) and stable (ratio ≤ 1.1; hyperplasia ≤ 10%) groups. The progression group included patients with progression of mesangial hyperplasia in the second biopsy specimen, whereas the stable group included patients with mesangial lesions that remained unchanged or showed regression in the second biopsy specimen. **Figure 1** depicts four light microscopy images of one patient each from the progression and stable groups. The biopsy specimens were evaluated by a single pathologist blinded to the clinical parameters of patients.

### Immunohistochemistry of RAS receptors and fibrosis-related indicators in the mesangium

Paraffin tissue sections were treated with primary antibodies (Abcam, Cambridge, UK) including collagen IV (Col IV; ab236640), fibronectin (FN; ab268020), laminin (LN; ab11575), anti-AT1R antibody (ab124505), anti-AT2R antibody (ab19134), anti-MasR antibody (ab66030), and anti-MrgD (ab18906).

We performed a semi-quantitative assessment of the intensity of immunohistochemical deposits. Normal tissues located far away from the tumor were used as normal controls. Image-Pro Plus software (Media Cybernetics, Bethesda, MD, USA) was used to analyze the pixel density of the stained areas and quantify the protein levels. The average optical density (AOD) was obtained by semi-quantitatively analysis of ten fields in a blinded manner.

### Statistical analysis

Statistical analyses were performed using SPSS software (version 25.0; IBM Corp., Armonk, NY, USA) and GraphPad Prism (version 8; GraphPad Software, San Diego, CA, USA). Continuous variables are presented as medians with interquartile ranges and were compared using the Mann-Whitney U test. Categorical variables are presented as counts (n) with percentages (%). The rate of eGFR decline from the time of second biopsy to the final follow-up was calculated by dividing the difference between the two time points by time. The p < 0.05 was considered statistically significant.

## Results

### Clinical variables at the time of biopsies

Of the 24 IgAN patients (17 males and 7 females), 12 and 12 were in the progressive and stable groups, respectively. The median ages of IgAN patients in the progressive and stable groups were 34 (20–45) and 34 (24–38) years, respectively (**Table 1**). The median interval between the renal biopsies was 4.3 (1–6) years. At the time of the first renal biopsy, there was no significant difference in the levels of eGFR or proteinuria between the progressive and stable groups (eGFR, 79.6 vs. 71.1 ml/min/1.73 m^2^; p = 0.55 and proteinuria, 3.3 vs. 3.1 g/24 h; p = 0.32). Similar results were observed at the time of the second biopsy. At the time of the second renal biopsy, there was a significant decrease in renal function compared to baseline values in the progressive (79.6 vs. 47.2 ml/min/1.73 m^2^, p < 0.01) and stable (71.1 vs. 46.8 ml/min/1.73 m^2^, p < 0.01) groups. Additionally, at the time of diagnosis, the progression group had a significantly lower serum albumin level than the stable group (39.5 vs. 44.5 g/L; p = 0.039). Furthermore, the proportion of use of RAS blockers after the first renal biopsy was significantly lower in the progression than stable group (41.7% vs. 83.3%; p = 0.035).

**Table 1:** Clinical data of patients with IgA nephropathy at the time of first and second renal biopsies.

	Progressive (n = 12)		Stable (n = 12)	
	1st biopsy	2nd biopsy	1st biopsy	2nd biopsy
Age, years	34 (20–45)	40 (25–54)	34 (24–38)	39 (30–51)
Males, n (%)	8 (66.7%)	–	9 (75%)	–
MAP, mmHg	92.5 (87.3–111.8)	100 (97–108)	108 (96–125)	94 (83.6–119.8)
BMI, kg/m^2^	23.9 (19.6–25.1)	23.8 (19.6–25.2)	24.1 (19.9–25.9)	24.0 (18.7–24.5)
Hemoglobin, g/L	136 (119–147)	120 (106–146)	127 (111–146)	127 (108–150)
Scr, µmol/L	99.2 (67.1–123.1)	167 (104–210)	116 (75–141)	154 (125–237)
Serum albumin, g/L	39.5 (27.2–42.0)^a^	41 (34–44)	44.5 (33.5–48.8)	38.5 (32.2–44.4)
Hematuria, n (%)	10 (83.3)	8 (66.7)	11 (91.7)	6 (50)
Proteinuria, g/24h	3.3 (1.6–3.7)	3.9 (3.4–6.8)	3.1 (1.1–3.6)	3.2 (1.4–4.5)
eGFR, ml/min/1.73 m^2^	79.6 (65.7–101)^b^	47.2 (23.9–70.8)	71.1 (51.8–108.6)^b^	46.8 (30.3–61.6)
Treatment after first biopsy				
RAS blockers, n (%)	5 (41.7)^a^		10 (83.3)	
Any immunosuppression, n (%)	6 (50.0)		9 (75.0)	

MAP, mean arterial pressure; BMI, body mass index; Scr, serum creatinine; eGFR, estimated glomerular filtration rate; RAS, renin angiotensin system. ^a^ p < 0.05 vs. the stable group. ^b^ p < 0.01 vs. same group.

### Deposition of RAS receptors between the first and second biopsies

**Figure 2:**
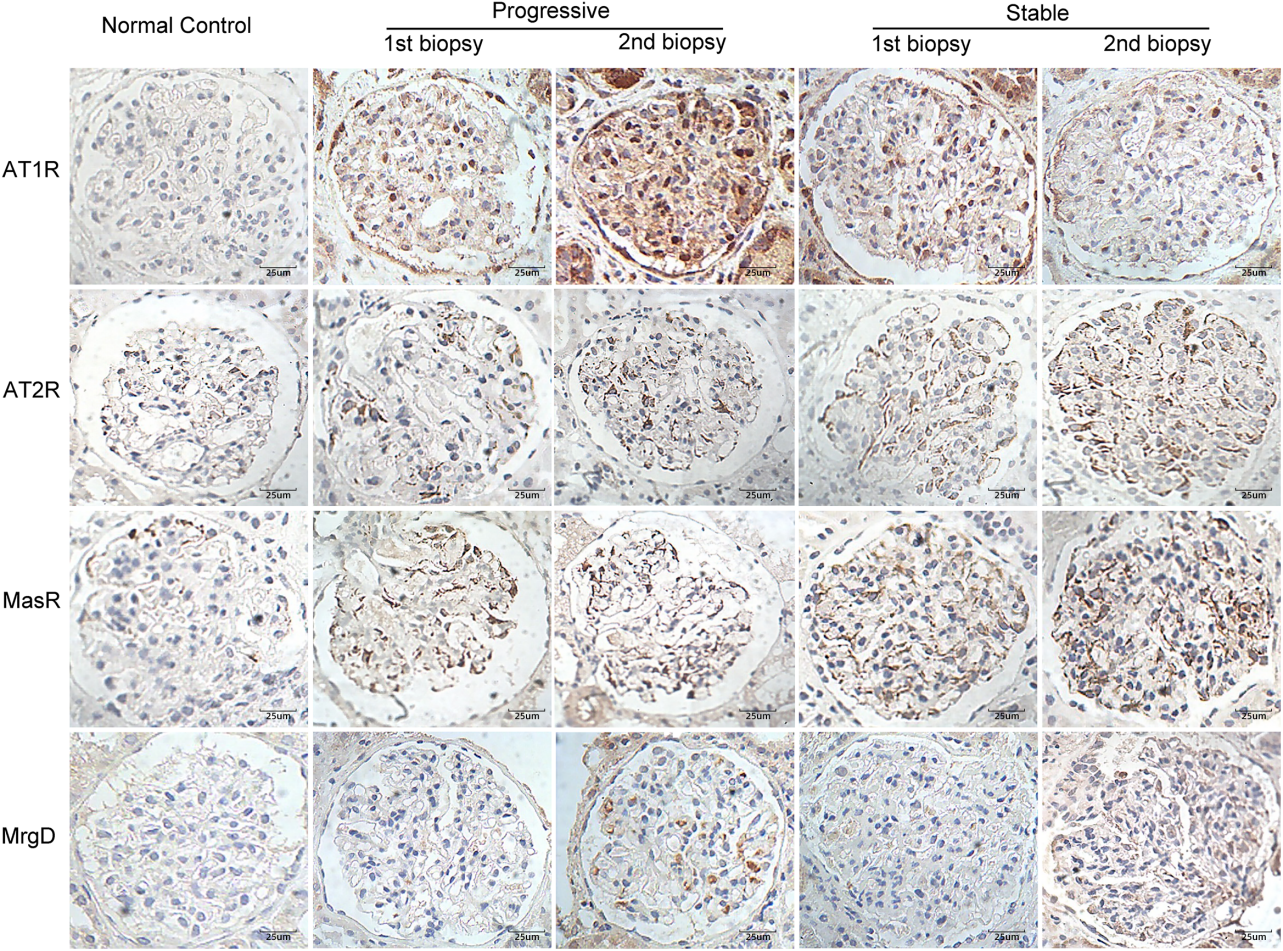
Immunohistochemical staining for AT1R, AT2R, MasR, and MgrD in patients with IgA nephropathy in the stable and progressive groups (×400). AT1R, angiotensin II type 1 receptor; AT2R, angiotensin II type 2 receptor; MasR, Mas receptor; MgrD, Mas-related G protein-coupled receptor, member D.

**Figure 3:**
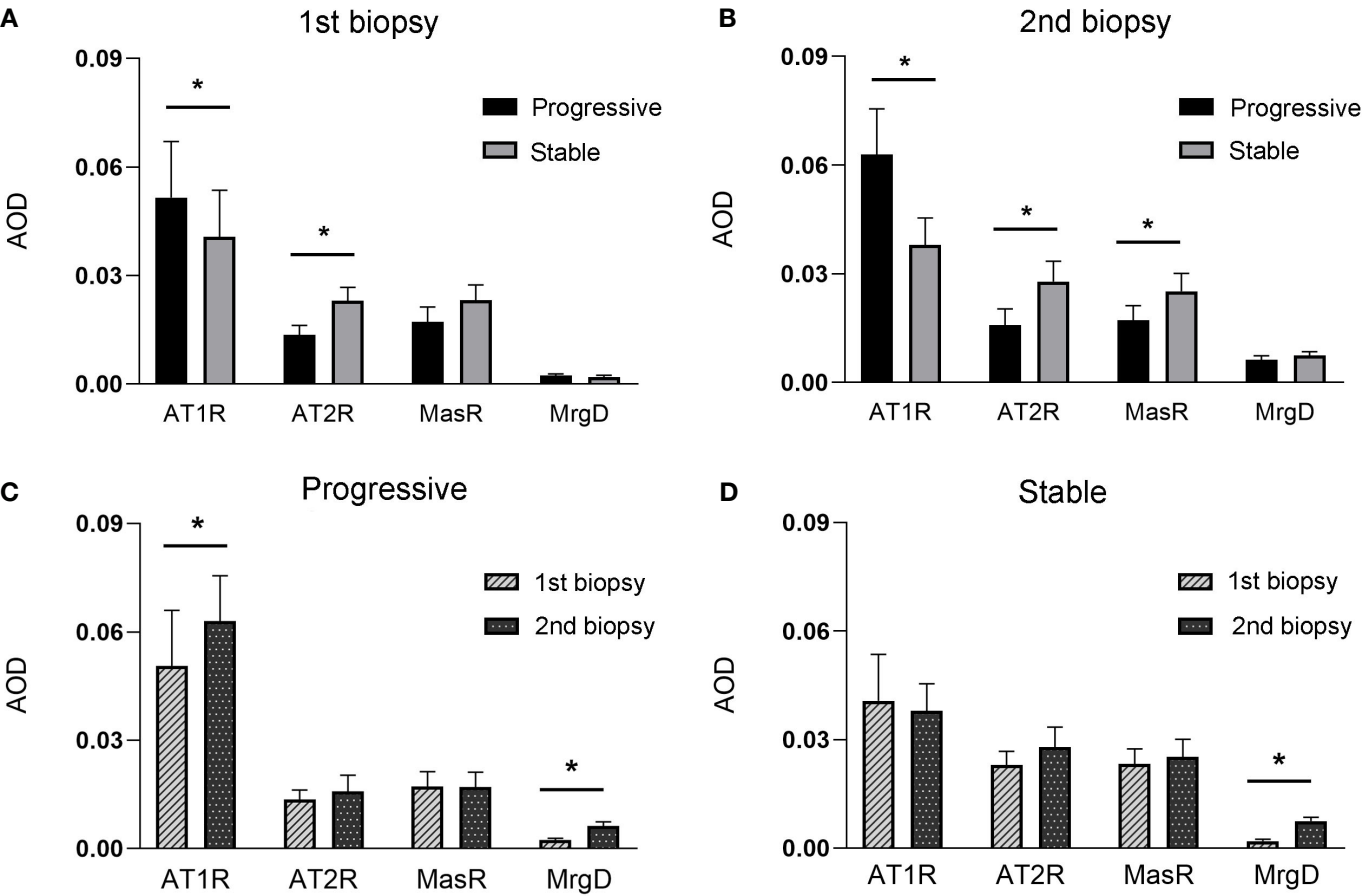
**(A, B)**, indicates the expression of renin-angiotensin (RAS) receptors between stable and progressive groups at the first **(A)**, or second **(B)** renal biopsy. **(C, D)**, indicates the expression of RAS receptors between the first and second biopsies in the progressive **(C)** or stable **(D)** groups.

**Figure 2** shows the immunohistochemical staining results for AT1R, AT2R, MasR, and MgrD in the glomeruli of patients with IgAN in the stable and progressive groups. As shown in **Figures 3A, B**, patients in the progression group had significantly higher expression of AT1R and significantly lower expression of AT2R at the time of the first and second renal biopsies (both p < 0.05). Patients in the progression group had significantly higher expression of MasR in the second biopsy specimen compared to the stable group. There was no difference in MrgD expression between the two groups in the first or second biopsy specimens. However, in both groups, the expression of MrgD was significantly higher in the second renal biopsy specimen compared to the first renal biopsy specimen, while the AT2R and MasR expression levels did not differ significantly between the first and second biopsy specimens (**Figures 3C, D**). The AT1R expression in the progression group was significantly higher in the second than first biopsy specimen. However, there was no difference between the specimens in the stable group.

### Deposition of Col IV, FN, and LN in the first and second biopsy specimens

**Figure 4:**
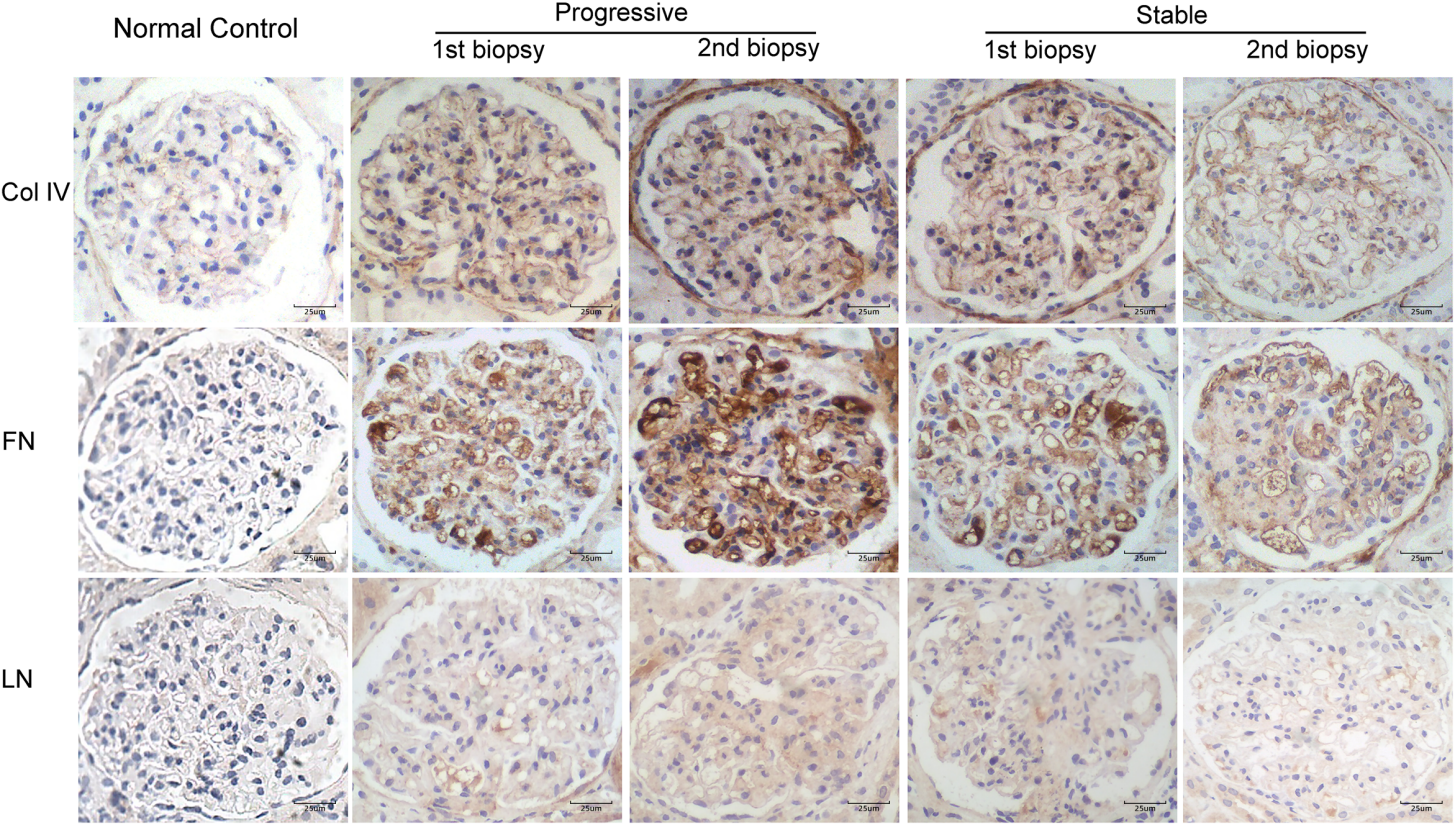
Immunohistochemical staining for Col IV, FN, and LN in patients with IgA nephropathy in the stable and progressive groups (×400). Col IV, collagen IV; FN, fibronectin; LN, laminin.

**Figure 5:**
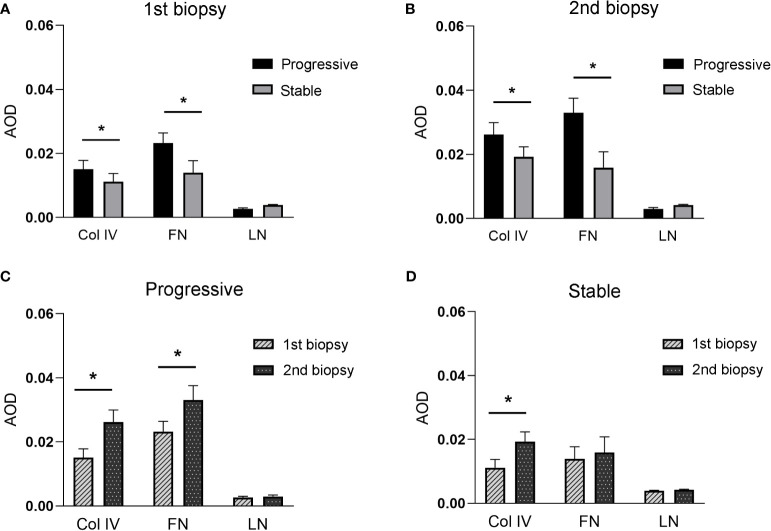
**(A, B)**, indicates the expression of Col IV, FN, and LN between stable and progressive groups at the first **(A)** or second **(B)** renal biopsy. **(C, D)**, indicates the expression of Col IV, FN, and LN between the first and second biopsies in the progressive **(C)** or stable **(D)** groups.

**Figure 4** displays the immunohistochemical staining results for Col IV, FN, and LN in the glomeruli of patients with IgAN in the stable and progressive groups. **Figure 5** presents the expression levels of COL IV, FN, and LN in the stable and progression groups. In the first biopsy specimen, the expression levels of mesangial matric proteins (COL IV and FN) were significantly higher in the progression than stable group. Patients in the progression group also had significantly greater Col IV and FN expression levels in the second than first biopsy specimen, suggesting progression of mesangial lesions (**Figures 5A, B**). The expression of COL IV in the stable group was also higher in the second than first biopsy specimen (**Figures 5C, D**).

### Clinical parameters predicting the progression of mesangial lesions in IgAN

**Table 2** shows the results of univariate and multivariate Cox regression analyses of the clinical parameters after the first renal biopsy, and the outcomes of mesangial lesions. The multivariate analysis showed a significant correlation between use of RAS blockers and the outcome of mesangial lesions (hazard ratio [HR], 0.27; 95% confidence interval [CI], 0.08–0.97; p < 0.05). The level of proteinuria at the time of the first renal biopsy also predicted the progression of mesangial lesions (HR, 1.8; 95% CI, 1.04–3.12; p < 0.05).

**Table 2:** Univariate and multivariate Cox analyses of clinical parameters predicting the progression of mesangial lesions in IgA nephropathy.

	Univariate analysis		Multivariate analysis	
	HR (95% CI)	p	HR (95% CI)	p
Age, years	0.95 (0.88–1.02)	0.16		
Male sex	0.86 (0.23–3.27)	0.82		
MAP, mmHg	0.99 (0.96–1.02)	0.61		
BMI, kg/m^2^	0.82 (0.62–1.06)	0.13		
Hemoglobin, g/L	1.0 (0.97–1.03)	0.89		
Serum albumin, g/L	0.95 (0.89–1.01)	0.12		
Hematuria, n (%)	0.38 (0.07–1.97)	0.25		
Proteinuria, g/24h	1.65 (0.99–2.76)	0.06	1.8 (1.04–3.12)	0.036
eGFR, ml/min/1.73 m^2^	1.01 (0.98–1.03)	0.63		
RAS blockers	0.33 (0.1–1.12)	0.08	0.27 (0.08–0.97)	0.045
Any immunosuppression	1.05 (0.31–3.54)	0.93		

MAP, mean arterial pressure; BMI, body mass index; eGFR, estimated glomerular filtration rate; RAS, renin angiotensin system; HR, hazard ratio; CI, confidence interval.

### Follow-up after the second biopsy

The median follow-up interval after the second renal biopsy in the 24 IgAN patients was 39.4 (21.0–58.3) months. **Table 3** presents the mean rate of eGFR decrease for the progression and stable groups. The rate of eGFR decline was significantly more rapid in patients with the progressive mesangial hyperplasia lesion than in those who had a maintained or reversal mesangial lesion (0.38 vs. 0.012 ml/min/1.73 m^2^/month, p = 0.004).

**Table 3:** Change in eGFR over time in IgA nephropathy patients in the progression and stable groups^*^.

	Progressive group	Stable group	p
Median follow-up, months	53.4 (25.7–57.2)	31.2 (17.6–77.5)	–
Differences between the initial and final values			
Change in eGFR, ml/min/1.73 m^2^/month	0.38 (0.04–1.02)	0.012 (-1.05–0.19)	0.004

eGFR, estimated glomerular filtration rate. ^*^Follow-up after the second renal biopsy.

## Discussion

In the present study, we analyzed the histopathological findings of repeat renal biopsies of IgAN patients. The progression of mesangial lesions was considered the outcome event. We compared clinical manifestations, drug therapy, and angiotensin receptor expression in renal specimen tissues between the progression and stable groups to identify factors related to the progression of mesangial lesions in IgAN. We confirmed the previously described discordance between the clinical and pathological features of repeat renal biopsies. Overexpression of intrarenal AT1R and massive proteinuria was associated with histological mesangial expansion. Sustained mesangial expansion was also associated with long-term renal impairment.

IgAN is the main disease leading to end-stage kidney disease. Glomerular mesangial lesions in IgAN are a direct cause of glomerulosclerosis, which may progress to renal failure. It is essential to develop strategies to prevent and treat glomerular mesangial lesions in IgAN. We therefore explored factors associated with the progression of mesangial lesions in IgAN patients who underwent two renal biopsies, to provide a basis for IgAN treatment. In the present study, the reasons for performing a second renal biopsy were treatment for more than one year but continuous unrelieved proteinuria, presence of acute renal injury clinically suspected to related to drugs, massive proteinuria that reappears after treatment, and so on. To avoid any effect of advanced age on glomerulosclerosis, we selected IgAN patients aged < 60 years at the time of the second renal biopsy with an interval of 1–6 years between the two biopsies. We excluded patients with prolonged disease duration to rule out any effect of disease duration on the progression of mesangial lesions.

Previous studies have found that pathologic data obtained from the second biopsy specimens may predict the development of end-stage kidney disease (14, 15). RAS blockers are useful for the treatment of mesangial proliferation (16–18). However, in the present study, some patients with persistently high AT1R expression were identified. Importantly, our findings may aid accurate classification and diagnosis of initial renal biopsy specimens from patients with non-genetic renal diseases, which may facilitate the selection of personalized therapy.

The RAS performs various functions in human physiology, particularly in the cardiovascular, respiratory, and renal systems (4). Activation of the intrarenal RAS stimulates mesangial cell proliferation and promotes mesangial matrix accumulation. AT1R is a key receptor driving disease progression. The molecular and cellular actions of angiotensin II in renal diseases are almost exclusively mediated by A1TR (19). In the present study, immunohistochemical analysis was used to detect RAS receptor expression in renal specimens of IgAN patients. The expression of glomerular AT1R was significantly higher in the progression than stable group, in both the first and second renal biopsy specimens. Compared to the first biopsy specimen, AT1R expression in the progression group was significantly higher in the second biopsy specimen, while there was no difference between the first and second biopsy specimens in the stable group. Multivariate analysis showed that a RAS inhibitor was protective against mesangial progression in IgAN patients, suggesting that glomerular AT1R is important for the reproduction of mesangial cells. Furthermore, inhibition of intrarenal RAS may prevent the progression of mesangial lesions, because angiotensin II can stimulate hypertrophy and the division of mesangial cell (5, 20).

AT1R, AT2R, MasR, and MrgD are the four main receptors of angiotensin. Previous studies have shown that AT1R mediates angiotensin II activity by activating several signaling pathways, such as the Wnt/β-catenin and TGF-β1-p53 pathways. The activation of these pathways is associated with vasoconstriction, renal fibrosis, and renal water and sodium absorption. AT2R prevents cell growth, and induces apoptosis and a renal autacoid cascade of vasodilator and natriuretic effects (20–22). Ang-(1–7) is a vasodilator and antiproliferative agent that binds to MasR and is produced by the hydrolysis of Ang II. Alamandine is a part of the alternative RAS and modulates MrgD expression through the PCK/ROS signaling pathway, which is a promising new drug target for kidney and heart protection (6, 7, 23, 24). In the present study, AT2R expression was significantly lower in the progression than stable group at the baseline and follow-up visits. MasR expression was higher in the stable than progression group only in the second biopsy specimen. However, no difference was found in MrgD expression between the progression and stable groups, in the first or second biopsy specimen. The expression levels of the aforementioned receptors were significantly lower than that of AT1R, suggesting that AT1R plays a major role in the progression of mesangial lesions. Even if other receptors are involved, it is likely that their roles are not sufficient to prevent the lesion progression caused by AT1R activation. Therefore, RAS receptors play a significant role in the progression of mesangial lesions in IgAN.

Mesangial matrix is produced by mesangial cells and consists mainly of Col IV, FN, and LN (25–28). Mesangial expansion is characterized by the accumulation of these components in the extracellular matrix. In the present study, we observed mesangial cell proliferation and increased expression of mesangial matrix proteins (Col IV and FN) in IgAN patients with progression of mesangial lesions compared to those with stable lesions. Patients in the progression group had significantly greater accumulation of Col IV and FN in the mesangial region in the second than first biopsy specimen, suggesting progression of the mesangial lesions.

We did not identify any effect of the baseline eGFR level on the progression of mesangial lesions. We then explored whether the histological progression of mesangial lesions has an effect on the decline in renal function after the second biopsy. Patients in the progression group exhibited more rapid decline in renal function compared to the stable group during the follow-up, which may be because of the short interval between the two biopsies and insignificant change in renal function. The progression of mesangial lesions exacerbates glomerular sclerosis, leading to deterioration of renal function.

We also observed that the level of proteinuria at the time of the first biopsy predicted the progression of mesangial lesions. Excessive proteinuria is often a manifestation of glomerular lesions. Severe mesangial expansion damages the glomerular basement membrane, thereby allowing the entry of macromolecular proteins into the glomeruli and excretion into the urine. Our findings provide additional evidence regarding the usefulness of repeat renal biopsy for guiding decision-making regarding subsequent treatment. We do not advocate the replacement of initial prognostic biopsy with the repeat biopsy; rather, we believe that both are important.

This study had several limitations. First, it was a single-center retrospective study with a small sample. Second, we did not include patients with other classes of renal pathological indexes, and only included patients with changes in the mesangial area. The M1 stage of the Oxford classification was used for the diagnosis of mesangial proliferation. Third, 62.5% of participants in this study used immunosuppressive therapy, and the median interval from the last dose of immunosuppression to the second biopsy was 3 (1.1–5) years. As the patients who underwent repeat renal biopsies in the present study was a skewed population of patients, they may not represent those with IgAN in the general population. Finally, the study patients underwent renal biopsies at different times during the natural history of IgAN. To minimize the effect of a prolonged disease course on the progression of mesangial lesions in IgAN, we only included patients who underwent a repeat renal biopsy after an interval of 1 to 6 years.

## Conclusion

In summary, continuous activation of the intrarenal RAS and high level of proteinuria correlate with the progression of mesangial lesions in IgAN patients. Accelerated progression of mesangial lesions may lead to premature renal failure. Our results highlight the usefulness of repeat biopsies for treatment evaluation.

## Data availability statement

The original contributions presented in the study are included in the article. Further inquiries can be directed to the corresponding authors.

## Ethics statement

The studies involving human participants were reviewed and approved by Ethics Committee of China-Japan Friendship Hospital. Written informed consent for participation was not required for this study in accordance with the national legislation and the institutional requirements.

## Author contributions

WL contributed to conception and design of the study. YL and SJ wrote the first draft of the manuscript. YL collected the clinical data. HG were responsible for histopathological preparation and staining. WL, YY, and SJ revised the final version. XL and WL were the guarantor of this work. All authors contributed to the article and approved the submitted version.

## Funding

This work was supported by grant from National Key Clinical Specialty Capacity Building Project (2019-542).

## Conflict of interest

The authors declare that the research was conducted in the absence of any commercial or financial relationships that could be construed as a potential conflict of interest.

## Publisher’s note

All claims expressed in this article are solely those of the authors and do not necessarily represent those of their affiliated organizations, or those of the publisher, the editors and the reviewers. Any product that may be evaluated in this article, or claim that may be made by its manufacturer, is not guaranteed or endorsed by the publisher.
